# Surface EMG-Based Instantaneous Hand Gesture Recognition Using Convolutional Neural Network with the Transfer Learning Method

**DOI:** 10.3390/s21072540

**Published:** 2021-04-05

**Authors:** Zhipeng Yu, Jianghai Zhao, Yucheng Wang, Linglong He, Shaonan Wang

**Affiliations:** 1 Hefei Institutes of Physical Science, Chinese Academy of Sciences, Hefei 230031, China; pengyz@mail.ustc.edu.cn (Z.Y.); ycwang@iamt.ac.cn (Y.W.); wangsnnn@mail.ustc.edu.cn (S.W.); 2 University of Science and Technology of China, Hefei 230026, China; hell616@mail.ustc.edu.cn

**Keywords:** transfer learning, instantaneous gesture recognition, surface electromyography, convolutional neural network

## Abstract

In recent years, surface electromyography (sEMG)-based human–computer interaction has been developed to improve the quality of life for people. Gesture recognition based on the instantaneous values of sEMG has the advantages of accurate prediction and low latency. However, the low generalization ability of the hand gesture recognition method limits its application to new subjects and new hand gestures, and brings a heavy training burden. For this reason, based on a convolutional neural network, a transfer learning (TL) strategy for instantaneous gesture recognition is proposed to improve the generalization performance of the target network. CapgMyo and NinaPro DB1 are used to evaluate the validity of our proposed strategy. Compared with the non-transfer learning (non-TL) strategy, our proposed strategy improves the average accuracy of new subject and new gesture recognition by 18.7% and 8.74%, respectively, when up to three repeated gestures are employed. The TL strategy reduces the training time by a factor of three. Experiments verify the transferability of spatial features and the validity of the proposed strategy in improving the recognition accuracy of new subjects and new gestures, and reducing the training burden. The proposed TL strategy provides an effective way of improving the generalization ability of the gesture recognition system.

## 1. Introduction

Human–computer interaction [1] can be leveraged to enable robotics following a person’s intentions [2,3,4], and enhance the capability of an individual collaborating with a machine, such as a disabled person wearing a prosthesis [5] to perform autonomous movement, as well as people wearing an exoskeleton [6,7] to carry a heavy load and save energy. In the context of wearable equipment control, myoelectric interfaces have the advantage of providing intuitive muscle activity. One of the important parts of myoelectric control [8,9,10] is technology that recognizes body gestures by surface electromyography (sEMG), which utilizes the non-invasive measurement of muscle activity to realize intention control of robotics. sEMG-based gesture recognition that decides the performance of myoelectric control has received much attention over the last decades.

Existing gesture recognition methods based on sEMG usually segment the sequence of myoelectric signals by sliding windows, and then extract descriptive features from the data segments [11,12,13]. Deep learning methods and traditional pattern recognition algorithms often take handcrafted features for gesture recognition. However, the accuracy of these algorithms are highly dependent on the reasonable handcrafted feature design [14,15]. In addition, for a myoelectric control system, the sEMG signals, representing the superpose of motor unit action potentials which are affected by the physiological state of the subject, are non-stationary [16]. Therefore, the sEMG recordings across subjects are user-dependent, and handcrafted features extracted from sEMG are also limited in terms of their generalization ability. Even under a user-dependent condition, the end goal remains the maintenance of accurate predictions, which needs a large amount of user data available and brings a heavy training burden to the system and users.

Reference [17] pays attention to instantaneous gesture recognition that takes raw sEMG as the input, proposes a recognition method with very little observation latency, and demonstrates that gesture patterns can be hidden inside the instantaneous values of sEMG. The end-to-end deep learning framework with the ability to automatically extract features provides a solution to reduce the limitation of feature design. Although user-dependent recognition exhibiting low generalization, the necessary condition to learn general feature mapping of sEMG may be fullfilled when mass data of multiple subjects are aggregated by history recording. This general feature mapping then facilitates hand gesture recognition for new subjects or even new gestures. Transfer learning [18] that was proposed to make use of the source domain knowledge can improve the performance of the discriminator in the target domain, providing inspiration to overcome the shortcoming of the training burden. Despite the advantages of instantaneous gesture recognition, including low prediction delay and accurate prediction, the transferability of the spatial features of instantaneous sEMG values has not been studied yet.

As such, to solve the problems of a heavy training burden and inaccurate predictions, this paper presents a novel transfer learning (TL) strategy to leverage source knowledge based on a convolutional network. We extract the spatial information of the gesture patterns from the instantaneous values of sEMG, which enables gesture recognition to be performed at a specific instant. The spatial pattern is composed of spatial features. The main hypothesis of our proposed TL method is that the spatial patterns of instantaneous sEMG between subjects are similar, and the spatial features between gestures are universal.

In the proposed TL framework, instantaneous raw sEMG data are taken as the input. The TL strategy first obtains the source spatial feature extractor by pre-training based on the source data and, then transfers the source spatial feature extractor to the target network. Finally, it fine-tunes the fully connected layers based on the target database. The major voting scheme is adopted to improve the recognition accuracy in the target domain. In this study, the target domain refers to new subjects and new gestures. To fully explore and verify the validity of our proposed method in the two transfer learning tasks, an evaluation was performed based on both the high-density sEMG(HD-sEMG) database [19] and sparse multi-channel sEMG database [20]. The main contributions of this paper can be summarized as follows: (1) We find the transferability of the spatial pattern hidden inside the instantaneous values of sEMG for new subject recognition; (2) We analyze the generalization ability of spatial features for new gesture recognition through the TL method.

This paper is organized as follows—an overview of the related works on sEMG-based pattern recognition using deep learning and transfer learning is given in Section 2. Section 3 thoroughly describes the structure of the instantaneous hand gesture recognition neural network and the TL strategy. Section 4 presents the evaluation databases and the experimental process, and the evaluation criteria are also included. Moreover, the experimental results and analysis are given in Section 5. Discussions of this work and indication of the possible direction for future research are presented in Section 6. Finally, conclusions are given in Section 7.

## 2. Related Works

In recent years, deep learning has attracted wide attention due to the powerful ability to capture the laws behind massive amounts of data. Deep learning can automatically extract information from the input, and allowing engineers and researchers to establish new understanding of objectives. It has been well-applied in many fields, such as computer vision and natural language processing.

In the field of gesture recognition, many researchers built the gesture recognition network based on handcrafted or non-handcrafted features. At present, the handcrafted features for gesture recognition are mainly designed in the time domain and frequency domain. Zhang [21] took five temporal features as the input and realized real-time gesture recognition based on the artificial neural network. Cote-Allard [22] combined the frequency features of sEMG into a graph, and fed the graph to the convolutional neural network. Chen [23] optimized the structure of the network proposed by Cote-Allard [22] to reduce the training parameters. Li [24] used S-transform to obtain the time-frequency joint features, and recognized hand gestures with four channels of sEMG.

In addition to the handcrafted features, reseachers [25,26,27,28,29] tried to use sequences of sEMG signals as the input. Park [25] applied deep learning to sEMG-based hand gesture recognition using a convolutional neural network. Atzori [26] compared the simply designed convolutional neural network architecture with classical classification methods given the same feature set, and the results showed the higher accuracy of the convolutional neural network. Ding [27] proposed a multiple-scale convolution structure that fully considers the characteristics of the sEMG signals, and experiments proved that the accuracy of the multi-scale raw sEMG input was higher than that of the handcrafted feature-based method. Asif [28] investigated the effect of hyper-parameters on each hand gesture, providing a robust and stable hand gesture design scheme for the deep learning-based approach. Tao [30] combined the IMU signal and the sEMG signal to improve the accuracy of gesture recognition. Further study [31] explained the ability of a convolutional neural network to combine data from two different modalities. Zhang [29] proposed a recurrent neural network-based approach that takes a window of sequential sEMG as the input, and the model could output predictions for the past sampling step in the window. However, the more data the window contains, the longer the prediction delay. Geng [17] introduced a deep convolutional neural network which outputs instantaneous prediction by giving the instantaneous values of sEMG, and proved that there is a spatial pattern of hand gestures hiding inside the instantaneous values of sEMG. This enables gesture recognition to be performed with sEMG at a specific instant. Instantaneous hand gesture recognition has the advantages of not only producing accurate predictions, but also generating outputs with very little observation latency. Deep learning has been rapidly developed in the field of sEMG-based pattern recognition, and its stronger learning ability than the machine learning method has proven its efficacy in improving the accuracy of sEMG-based hand gesture recognition. However, few studies have focused on improving the generalization of the deep network and reducing the heavy training burden of the myoelectric control system.

As a branch of deep learning, transfer learning [32] discovers and leverages latent knowledge from source tasks to improve the prediction performance in the target domain. Cote-Allard [33] first proposed inter-subject recognition by adopting Progressive Neural Network (PNN) [34]. In their work, transfer learning was performed based on one repetition of seven gestures, and the target network achieved an average accuracy from 86.77% to 93.36%, which validated its ability to reduce the training burden. A further study by Cote-Allard [35] validated the improved PNN for the recognition of eleven new gestures, and the proposed method achieved a recognition accuracy of 49.41% compared to 46.06% without transfer learning. Chen [36] introduced an HD-sEMG based transfer learning method using a convolutional neural network. By pre-training based on a multi-mode dataset that contains 30 gestures, keeping some of the parameters consistent with the target network, and performing transfer learning based on two repetitions of training data for each gesture, the recognition accuracy of new subjects and 10 new gestures could reach 90%. The increased accuracy and decreased training time verified the significance of the convolutional neural network-based transfer learning in improving generalization and training burden reduction. However, the proposed method is restricted in an HD-sEMG scenario and the transfer learning performance is highly dependent on the specific database design, limiting its general usage.

To reduce the limitation of user-dependent recognition, Du [19] provided an HD-sEMG database named CapgMyo and proposed a multi-stream AdaBn domain adaptation method. With the adoption of the adaptation method, the new subject recognition accuracy for 12 gestures rose from 26.3% to 35.1%. Ketyko [37] proposed a 2-stage recurrent neural networks(2SRNN). The first stage pre-trained a recurrent neural network based on the source database, and the second stage learned a linear transformation matrix based on the target database. In their study, 50% of target subject data were used for second stage learning, while the accuracy of new subject recognition reached 91.1% and 65.1% for the high-density sEMG database and sparse multi-channel database, respectively. Kim [38] presented a novel subject-transfer framework which trains the source classifier for each source subject in a user-specific approach, and the prediction for hand gestures of the target subject was carried out by voting the output of the selected ten fine-tuned source classifiers. The experimental results displayed an improvement of the accuracy from 49.76% to 52.52%.

Although relevant studies on sEMG-based hand gesture recognition have verified that transfer learning can improve the generalization of the source network for new subjects or new gestures recognition to some degree, some issues, such as the heavy training burden [37], strict condition of use [36] and low generalization of the algorithm remain unsolved [19,35,38]. Yosinski [39] explored the transferability of features in the deep neural network, and the results proved that the transferability of features decreases when transferring a higher task-specific layer. In other words, shallow layers with transferred features have a better property of generalization in a new task. In addition, instantaneous hand gesture recognition has the advantages of low observation latency and a high recognition accuracy [17]. The good characteristic of instantaneous recognition and generalization ability of shallow layers inspired us to explore the possibility of transferring spatial information hidden in sEMG.

## 3. Instantaneous Hand Gesture Recognition Neural Network and TL Strategy

### 3.1. Input of Instantaneous Hand Gesture Recognition

**Figure 1:**
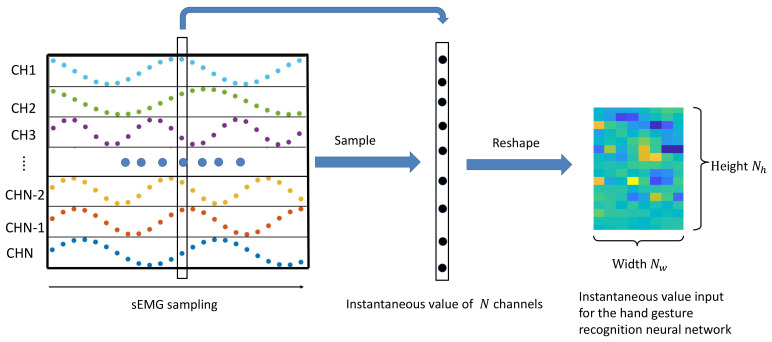
Input preparation process of the instantaneous gesture recognition network. CH denotes channel.

The input preparation process of the instantaneous gesture recognition network is shown in Figure 1. First, the sEMG signal with *N* channels is sampled to obtain a vector containing one frame of the instantaneous values of each channel, and then this *N*-dimensional vector is reshaped into a graph of size $N_{h}\times N_{w}$, where $N=N_{h}\times N_{w}$. Finally, we obtain the input for the instantaneous gesture recognition network. It is worth noting that after obtaining the sampled value of the signal, we use the raw EMG signal as the input to make a prediction for each sampling moment.

### 3.2. Structure of Instantaneous Hand Gesture Recognition Neural Network

**Figure 2:**
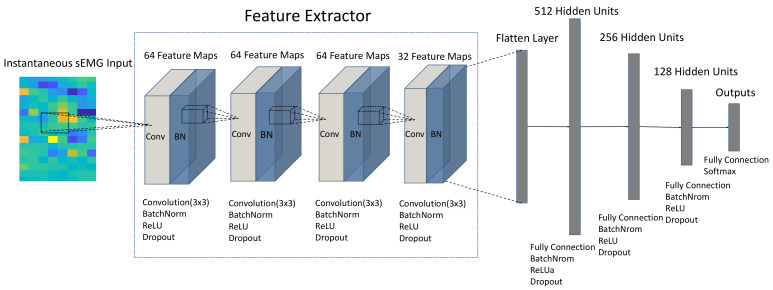
The structure of instantaneous hand gesture recognition neural network. Conv and BN denote Convolution and Batch Normalization, respectively.

In this section, we define the architecture of the instantaneous hand gesture recognition neural network based on a convolutional neural network, which explores the spatial information of instantaneous sEMG values and makes instantaneous prediction. As shown in Figure 2, the instantaneous recognition network contains a feature extractor and three fully connected layers. The predictions of hand gesture are obtained through a G-way fully connected layer and a softmax function, where G represents the category of gestures to be recognized. The feature extractor of the network is comprised of four convolution layers. The first three convolutional layers consist of 64 3 × 3 filters to capture the sEMG spatial features, and the last convolution layer has 32 filters to reduce the feature dimension. All convolution layers have a stride of 1 and padding of 1. The output of the last convolution layer is flattened into one-dimensional vector through a flatten layer. The three fully connected layers contain units of 512, 256, and 128, respectively. BatchNorm, ReLU and Dropout are subsequently considered after each layer. BatchNorm following the input and each layer is considered to accelerate training [40]. The ReLU no-linearity function is adopted to prevent gradient vanishing [41]. Dropout with a probability of 0.2 after each ReLU is used to avoid over-fitting [42]. In addition, weight decay with a rate of 0.001 is also treated as weight regularization and applied in each layer to improve the generalization of the neural networks [43].

### 3.3. TL Strategy

#### 3.3.1. Problem Definition

When we use the sEMG signals for gesture recognition, there are two common problems we have to face. First, it is challenging to recognize hand gestures of a new subject due to subject-dependent characteristic of sEMG. Second, it is difficult to train a new classifier with a high accuracy when only a small amount of target training data available [35,36,38]. It is important to have a high recognition accuracy of the classifier while only using a small number of gesture repetitions of new subjects and new gestures for training. Because it can reduce the user’s data collection burden, and thereby reducing the limitation of hand gesture recognition application. The spatial features of the instantaneous sEMG values may have the ability to improve the generalization performance of the gesture recognition network, which is need further study. In summary, we have two transfer learning tasks—verifying the generalization ability of the spatial features of the instantaneous sEMG values for the new subject and new gesture recognition.

#### 3.3.2. Target Network Training Strategy

**Figure 3:**
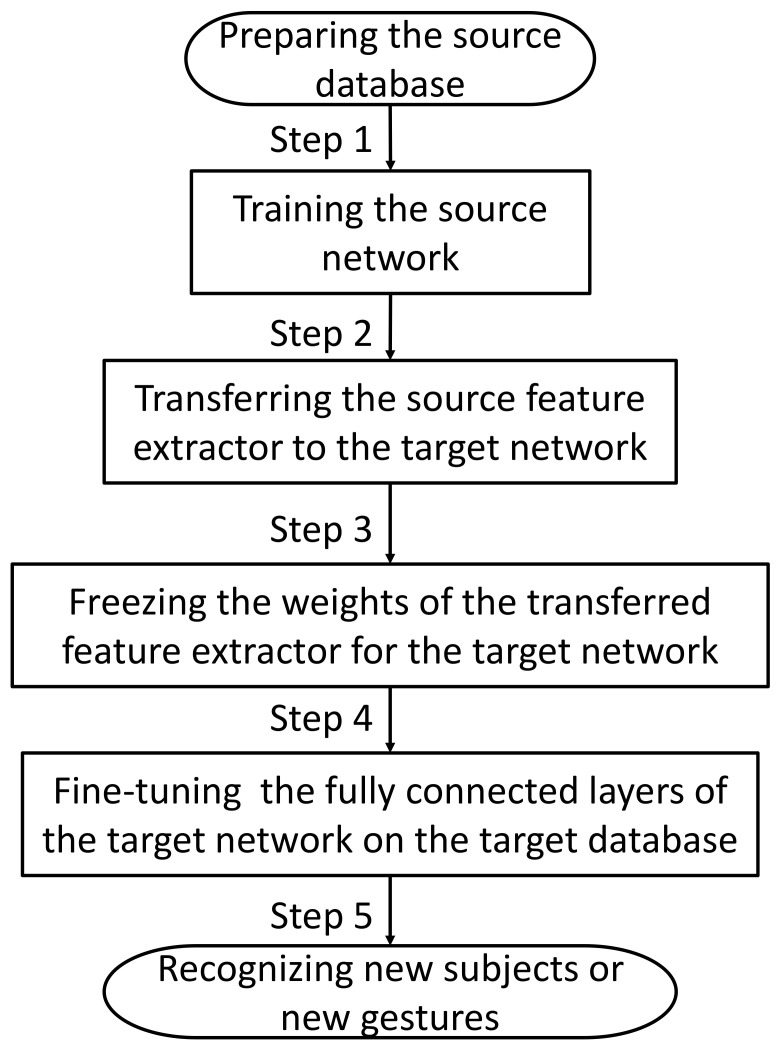
The training process of the target network.

Assuming that the well-trained instantaneous hand gesture recognition network can capture the representative feature of source gestures, for small-scale new subject data or new gestures in the target gesture set, transferring some source domain knowledge stored in the feature layer to the target network is expected to boost generalization and accelerate training. In this study, we keep the structure of the target network consistent with the source network. The target network training process is shown in Figure 3.

**Figure 4:**
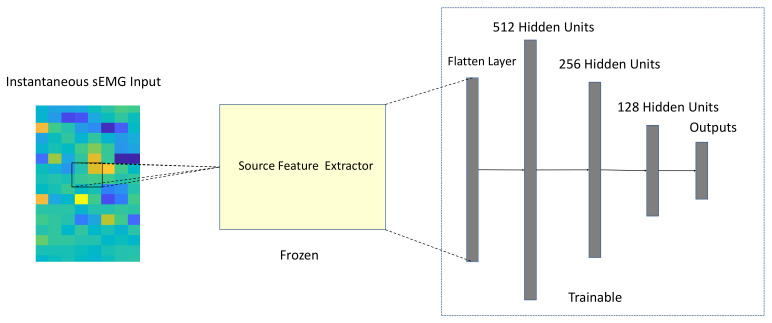
The architecture of the target network with the transfer learning (TL) strategy.

The first step is to train the source network on the source database. Then, in step two, the well-trained source domain feature extractor of the instantaneous classifier is transferred to the target network so that the target network could utilize source knowledge directly. In the third step, freezing the weights of the transferred feature extrator makes sure source knowledge retaining in the next step. Step four is to fine-tune the fully connected layers of the target network on the target database. And the last step is recognizing new subjects or new gestures by the target network. The architecture of the target network with the TL strategy is shown in Figure 4.

The non-transfer learning (non-TL) strategy uses the same structure of the target network, but the weights of the feature extractor is random initialized and trainable. And non-TL performed training directly on the target database.

#### 3.3.3. Post-Processing

Transfer learning makes use of source knowledge in the feature extractor, and fine-tunes the last three fully connected layers by using target date. If a small amount of target training data is available, the target network can capture spatial information about new subjects and new hand gestures. Additionally, the sEMG signal is time sequence data that measure muscle activation. To complete a hand gesture, a set of motor unit action potential occurs and the sEMG signal is composed successively [44]. It is common to assign the sEMG signal the same gesture label for one movement cycle [45]. Therefore, a simple major vote scheme could be used to enhance the prediction accuracy [46,47]. To get a final prediction label for the time window [t,t+m], we get *m* instantaneous prediction results p(s), where s∈[t,t+m]. The simple major vote scheme can be formed as

(1) $$P=\arg\max_{c}\left\{n_{1},\cdots n_{c},\cdots n_{G}\right\},where\;n_{c}=sum\left(w_{sc}p\left(s\right)\right)$$

(2) $$w_{sc}=\left\{\begin{array}{c} 1\;if\;p(s)=c\\ 0\;others, \end{array}\right.$$

where $n_{c}$ is the number of gestures predicted as *c*, and *P* is the major voted prediction gesture for instant s∈[t,t+m]. The simple major vote scheme is applied without window overlapping.

## 4. Experiment

### 4.1. Evaluation Dataset

**Figure 5:**
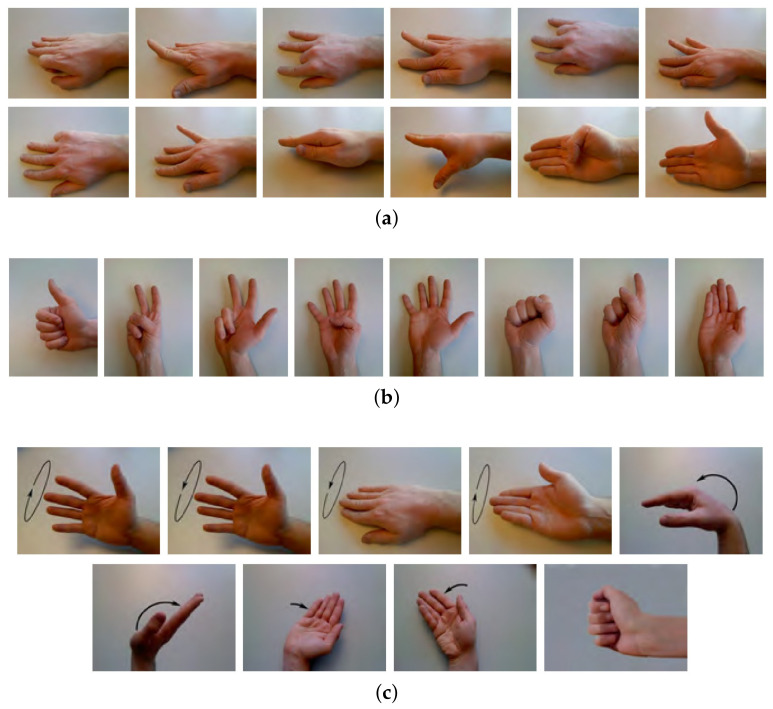
Finger and wrist gesture categories in CapgMyo [19] and NinaPro DB1 [20]. (**a**) Twelve basic movements of finger in DB-c and Exercise A; (**b**) eight isometric and isotonic hand configurations in DB-a and Exercise B; and (**c**) nine basic movements of the wrist in Exercise B.

Existing hand gesture recognition systems can be broadly divided into two categories according to their input: (1) systems based on the HD-sEMG input mode and (2) systems based on the sparse multi-channel sEMG input mode. To fully explore and evaluate our proposed method in two kinds of transfer learning task, we verified our TL method validity based both on the HD-sEMG database and sparse multi-channel sEMG database. Therefore, we recruited two public databases: (1) CapgMyo [19] and, (2) Ninapro-DB1 [20]. The first one is an HD-sEMG database, while the second one is a sparse multi-channel database. Figure 5 shows the states of finger and wrist gestures in CapgMyo and NinaPro DB1. The two databases contain a wealth of gesture types and high-quality data which have been preprocessed by acquisition devices, so that researchers can study algorithms directly. To use the two databases, we simply use and divide them into training set and test set.

CapgMyo is comprised of 128-channel HD-sEMG data sampled at 1000 Hz and contains three sub-databases. In this study, CapgMyo-DBa and CapgMyo-DBc (denoted as DB-a and DB-c, respectively) were chosen to evaluate new gestures and new subjects recognition. DB-a contains eight isometric and isotonic hand gestures (shown in Figure 5b) recoded from 18 subjects, while DB-c contains twelve basic movements (shown in Figure 5a) of fingers obtained from 10 intact participants. The gestures in both sub-databases are repeated 10 times.

NinaPro DB1 is a 10-channel sEMG database at a sampling rate of 100 Hz, and contains data from 27 participants. It was divided into three sub-databases, including-Exercise A, B, and C, which were composed of 12, 17, and 23 different movements, respectively. Each movement was recorded 10 times. Exercise A contains twelve basic movements of fingers. Exercise B contains eight isometric and isotonic hand configurations and nine basic movements of the wrist (shown in Figure 5c), for which the first eight hand gestures are consistent with DB-a. To evaluate the TL method, Exercise A and Exercise B were adopted.

The twelve basic movements of fingers make up different gesture configurations. The gestures become more complicated after adding the movement of the wrist. It is necessary to improve the generalization ability of the basic finger movement recognition among different subjects due to its commonly used in many sEMG-based applications [48,49,50]. The basic movements of fingers compose different gesture configurations and complex gestures, which may provide useful information to learning new gestures. Therefore, we recruit DB-c and Exercise A for new subject recognition experiments, and DB-a and Exercise B for new gesture recognition experiments.

### 4.2. Method of Training

In this study, we applied transfer learning to new subject recognition and new gesture recognition. To fully explore the validation of our proposed TL method, we evaluated the method in HD-sEMG input mode and sparse sEMG input mode. For DB-a and DB-c with 128 channels, the input shape of the instantaneous recognition network is set to 8 × 16. For Exercise A and Exercise B with 10 channels, the input shape is set to 1 × 10.

When considering the source database and target database construction, we treated the data of each person in DB-c and Exercise A in turn as a new subject to construct the target database, and the data of other subjects were used to construct the source database. For new gesture recognition, we used DB-c and Exercise A with the twelve basic finger movements to build the source database. The gesture data of each person in DB-a and Exercise B took turns to build the target database.

We divided the target database into a training set and a test set according the repetition number of each gesture. $N_{t}\left(1\leq N_{t}\leq 7\right)$ repetitions were randomly selected from each gesture of the target database to construct the training set, and the remaining repetitions were used for testing. Therefore, each target database can be divided into 7 training sets and test sets with different sample sizes.

During the training of the instantaneous hand gesture recognition neural network, adaptive moment estimation (Adam) [51] was selected as the network optimizer, with an initial learning rate of 0.0001. Mini-batch training was employed to prevent difficulty convergence that appeared in single batch training [52]. The pre-training epoch and transfer learning epoch were both set to 100. The batch size was set to 1000 for CapgMyo and 100 for NinaPro DB1. Computations of instantaneous recognition network were carried out on one Xeon 5122 CPU and one Titan Xp GPU.

The non-TL strategy used the same training set and test set of the TL method to fairly comparison.

### 4.3. Post-Processing

The power of the major vote strategy for hand gesture recognition has been verified in many studies [17,19,29]. According to these studies, the major vote scheme could evidently improve the recognition accuracy within a finite sample. However, there is no free lunch in the world. As the number of voting samples increases, the delay of prediction will also increase. In order to balance the delay and accuracy of the prediction, the number of voting samples was decided based on previous research [17]. For the CapgMyo database with 1000 Hz sample rate and NinaPro DB1 with 100 Hz sample rate, the voting number was set to 100 and 28, which introduced a prediction delay of 100 ms and 280 ms, respectively.

### 4.4. Evaluation Criteria and Statistical Analysis

In order to evaluate the effectiveness of the proposed TL strategy in improving the generalization ability of the target network and reducing the training burden, the recognition accuracy and training time were taken as the criteria. The statistical analysis of two-way ANOVA was used to quantitate the influence of the gesture repetition and training strategy on the training time, instantaneous recognition accuracy, and major voting accuracy of the target network. Multiple comparison was implemented to determine the performance variation among repetitions. The results presented as *p* < 0.05 were regarded as significant.

## 5. Results and Analysis

### 5.1. Transfer Learning for New Subject Recognition

**Figure 6:**
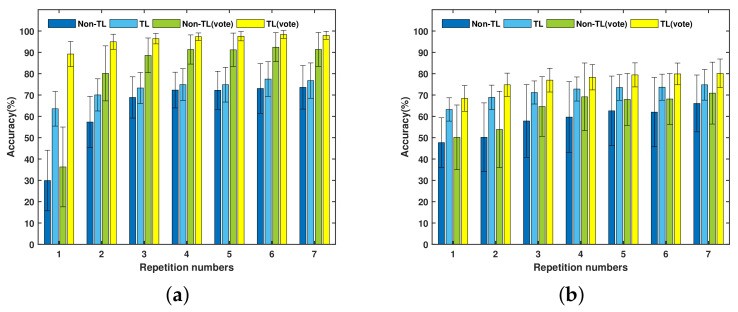
The comparison of the accuracy of instantaneous recognition and major voted recognition of the target network under the TL strategy and non-TL strategy for new subject recognition: (**a**) DB-c and (**b**) Exercise A.

Table 1 gives the significant influence of the TL strategy and repetition number through two-way ANOVA. * means the significant difference of *p* < 0.001. Figure 6 shows the average new subject recognition accuracy with standard deviations of two databases under two training strategies and two kinds of post-processing (instantaneous prediction and major voted prediction). Up to 7 repetitions are assigned to each subject in DB-c and Exercise A. Table 2 lists the average instantaneous accuracy and major voted accuracy for new subject recognition. Table 3 compares the average training time of new subject recognition under the TL strategy and non-TL strategy. From the results presented in Figure 6, Table 1, Table 2 and Table 3, the following points can be drawn.

First, the proposed TL strategy can significantly improve both the instantaneous recognition accuracy and major voted recognition accuracy. On the whole, the two recognition accuracies based on the TL strategy are higher than that with non-TL in DB-c and Exercise A. By training the target network with the TL strategy, the corresponding average instantaneous recognition accuracy of DB-c and Exercise A are 72.98 ± 4.46% and 71.16 ± 3.7%, respectively. In comparison, when training with the non-TL strategy, the average instantaneous recognition accuracy are 63.91 ± 14.8% and 58 ± 6%, respectively. For the TL strategy with major voted post-processing, the average recognition accuracy of DB-c and Exercise A are 95.97 ± 2.95% and 76.9 ± 3.8%, respectively. However, when training the target network with the non-TL strategy, the corresponding average major voted recognition accuracy of DB-c and Exercise A are 81.6 ± 18.9% and 63.4 ± 7.5%, respectively.

Second, the number of repetitions has a significant influence on both the instantaneous recognition accuracy and major voted recognition accuracy. Concretely, two kinds of post-preprocessing recognition accuracy increase as the number of repetitions increases. The result of post-comparison shows that the recognition accuracy of the first three repetitions has increased significantly. When the gesture data of more than four repetitions is obtained, the difference of accuracies between adjacent repetitions is not significant. Compared with the first three repetitions, the accuracy of the last four repetitions increases slowly. In other words, the instantaneous recognition accuracy and the major voted accuracy increase rapidly when a small number of repetitions are given. However, the accuracy of the TL strategy and non-TL strategy are different. As shown in Figure 6, the TL strategy can get better performance than non-TL strategy, especially if the gesture repetition is small. When at most three gesture repetitions are given, the TL strategy can improve the accuracy of DB-c and Exercise A by an average of 21.05% and 16.52%, respectively. This means that our TL strategy reached an average 18.7% improvement for the first three repetitions. For the TL strategy with only one repetition gesture, the instantaneous recognition accuracy is 62% for the two databases, while the major voted recognition accuracy of DB-c and Exercise A reach 89% and 68.4%, respectively. Given more than two repetition gestures, the instantaneous recognition accuracy is higher than 70%, while the major voted recognition accuracy can reach 95% for DB-c and 75% for Exercise A. For training conditions with the non-TL strategy, even the number of repetitions has more of an effect on it than that of the TL strategy, it is still difficult for the recognition accuracy of the non-TL strategy to surpass that of the TL strategy.

Third, the proposed TL strategy of the target network can significantly reduce the system training time. More specifically, the TL strategy reduced the average training time of the target network from 172.72 to 51.3 s and 72.33 to 24.32 s for DB-c and Exercise A, respectively. Gesture repetition has an influence (*p* = 0.029 for DB-c, *p* = 0.02 for Exercise A) on the training time for the two databases. For the TL strategy, increasing the number of training data could introduce a long training time. However, for training with the non-TL strategy, training with little repetitions and multiple dropouts also made the training time longer.

### 5.2. Transfer Learning for New Gesture Recognition

**Figure 7:**
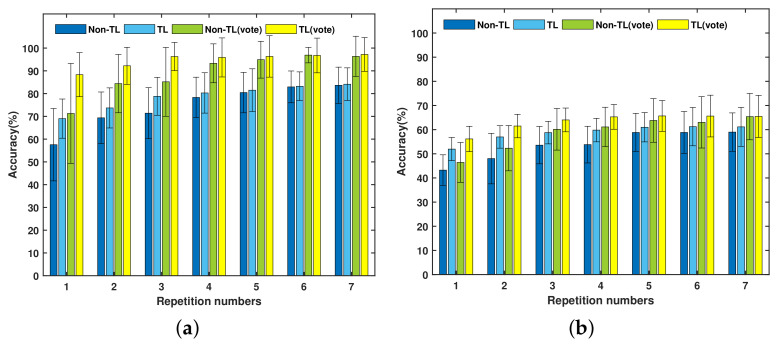
The comparison of the accuracy of instantaneous recognition and major voted recognition of the target network under the TL strategy and non-TL strategy for new gesture recognition: (**a**) DB-a and (**b**) Exercise B.

Figure 7 represents the average new gesture recognition accuracy of DB-a and Exercise B under two training strategies and two kinds of post-preprocessing. Table 4 lists the average instantaneous accuracy and major voted accuracy for new gesture recognition. Table 5 describes the average training time of new gesture recognition under the TL strategy and non-TL strategy. Two-way ANOVA has been employed to evaluate the significant influence. Statistical analysis results are given in Table 1. From the results obtained in Figure 7, Table 1, Table 4 and Table 5, the following points can be summarized.

First, the TL strategy can significantly improve the new gesture recognition of the instantaneous accuracy and major voted accuracy. On the whole, the two kinds of accuracy of the TL strategy are higher than those of the non-TL strategy for both the DB-a and Exercise B databases. By training the target network with the TL strategy, the average instantaneous recognition accuracy of DB-a and Exercise B are 78.7 ± 5% and 58.7 ± 3%, respectively. In comparison, when training with non-TL strategy, the average instantaneous recognition accuracy of DB-a and Exercise B are 74.8 ± 8.6% and 53.6 ± 5.6%, respectively. For training with major voted post-processing, the TL-based average accuracy of DB-a and Exercise B are respectively 94.7 ± 3% and 63.4 ± 3.2%. However, when training the target network with non-TL strategy, the corresponding average major voted recognition accuracy of DB-a and Exercise B are respectively 89.8 ± 8.6% and 58.9 ± 6.4%.

Second, the number of repetitions has a significant influence on the instantaneous accuracy and major voted accuracy of new gesture recognition. The two kinds of post-processing recognition accuracy increase as the number of repetitions increases. According to the results of post-comparison, the accuracy of the last four repetitions does not increase significantly, while the accuracy of the first three repetitions has a significant increase. Specifically, when maximum three gesture repetitions were given, the TL method improved the accuracy of DB-a and Exercise B by an average of 9.86% and 7.62%, respectively. This means that the proposed TL strategy reached average 8.74% improvement for the first three repetitions. However, the effects of repetition number on the TL and non-TL strategy for new gesture recognition are different in Figure 6. The TL strategy is less affected by gesture repetition. For TL with only one repetition gesture, the instantaneous recognition accuracies are respectively 69% and 52% for DB-a and Exercise B, while the average major voted accuracies are respectively 88.3% and 56.2%. When more than two repetition gestures are employed, the instantaneous recognition accuracy is higher than 70% for DB-a and 55% for Exercise B, while the major voted recognition accuracy reaches 95% and 62% for the two databases, respectively. For the training condition with the non-TL strategy, the recognition accuracy can approach that of the TL-based method after five repetitions in DB-a, but it still difficult to catch up in Exercise B.

Third, the proposed TL strategy of the target network can significantly reduce the system training time of new gesture recognition. Specifically, the TL strategy reduced the average training time from 92 to 23.3 s for DB-a and 120 to 44.96 s for Exercise B. In addition, two-way ANOVA revealed that gesture repetition has no significant effect on the training time for DB-a (*p* = 0.995) and Exercise B (*p* = 0.584). For DB-a under the TL strategy, although the training time increases slightly with the increase of the number of repetitions, it is still much lower than the required training time of non-TL.

## 6. Discussions

The sEMG-based control system suffers from the low generalization problem, which induces a heavy training burden for new subject and new gesture recognition. However, if the necessary condition for learning general feature mapping of sEMG is met, transfer learning can be used to improve the performance of new subject and new gesture recognition. In this study, the convolutional network takes instantaneous raw sEMG as the input, and transfer learning is recruited to evaluate the transferability of spatial features for new subject and new gesture recognition. The feasibility, validity, and limitations of the proposed TL strategy are discussed below. In addition, we make a comparison with related works, and a comparison for the HD-sEMG database and the sparse multi-channel database. Also, we include the discussion of prediction delay.

### 6.1. Feasibility of Transferring Spatial Features through the Proposed TL Strategy

The spatial information of the source domain is employed to improve the performance of the target network for new subject and new gesture recognition. Experiments of the two transfer learning scenarios have been conducted on both the HD-sEMG database and sparse multi-channel database. The experimental results indicate that the spatial information-based TL strategy has a positive influence on the myoelectric recognition system. Considering the two kinds of post-processing together, under the condition of training with non-TL strategy, the average accuracies of new subject recognition and new gesture recognition are 72.7% for DB-c, 61% for Exercise A, 81.85% for DB-a and 56.25% for Exercise B. When the TL strategy is adopted, the average accuracies of new subject and new gesture recognition are improved to 84.47% for DB-c, 74.7% for Exercise A, 86.7% for DB-a, and 61.5% for Exercise B. The high accuracy of the TL strategy for the four databases verify the transferability of the spatial pattern hidden inside the instantaneous values of sEMG and generalization ability of the spatial features. This excellent transferable property of spatial features makes new subject and new gesture recognition more accurate and makes it possible to build a low latency but high generality recognition system.

### 6.2. Validity of the Proposed TL Strategy in Reducing Training Burden for Hand Gesture Recognition System

The experimental results prove that significant ability of the TL strategy in reducing the training burden of new subject and new gesture recognition. Although the improvement of new subject and new gesture recognition is not obvious by using more than four repetitions for training, when only three repetition gestures can be used for training at most, the recognition accuracy is significant increased. This means the performance of the classifier could be significantly improved while using a small amount of training data, thereby reducing the training burden. More specifically, the training burden can be considered from user data collection and system training perspectives. The user data collection burden refers to the gesture repetition required for the target network training. The proposed TL strategy makes target network training less affected by gesture repetition, but retains the high recognition accuracy even given little repetition training data. With one repetition training data, the major voted recognition accuracies under the TL strategy are 72.98% for DB-c, 68.4% for Exercise A, 88.3% for DB-a, and 56.2% for Exercise B. When more than two repetitions are included, the major voted accuracies reach 95% for DB-c, 75% for Exercise A, 95% for DB-a, and 62% for Exercise B. However, for training with the non-TL strategy, it takes more than five repetitions for the target network to get a high recognition accuracy, but it is still difficult to catch up with the TL-based accuracy. The system training burden refers to the required training time of the target network. The TL strategy can significantly reduce the training time. The average training time is reduced from 172.7 to 51.3 s for DB-c, 72.33 to 24.32 s for Exercise A, 92 to 24.3 s for DB-a and 120 to 44.96 s for Exercise B. Compared with non-TL, the training time of the TL strategy has been reduced by a factor of three. The training time reduction is mainly due to the reduction of trainable parameters of the target network [53]. Non-trainable parameters are contained in the spatial feature extractor, which has been pre-trained based on the source database. Fine-tuning small parts of target network parameters can utilize small amounts of training data, but cost little training time and produce a high performance for the hand gesture recognition system. This advantage of fine-tuning is guaranteed by the generalization of spatial features and can actually improve the training burden.

### 6.3. Comparison with Related Transfer Learning Strategies

Related studies have been focused on leveraging source knowledge of gesture recognition to improve the performance of the prediction function in the target domain. Cote-Allard [33] first proposed inter-subject recognition by adopting PNN. Subsequently, Cote-Allard [35] improved the PNN obtaining a better performance. Evaluation of new subject recognition were performed on 8 channel sEMG database. By using the four repetition gestures of new subject, the average accuracy of new subject recognition were 58.41% for the TL-based strategy and 54% for the non-TL based strategy, and our proposed method obtained 74.62% for the TL-based strategy and 59.47% for the non-TL based strategy. Chen [36] introduced an HD-sEMG based transfer learning method using a convolutional neural network. By pre-training based on a 30 multi-mode gesture dataset, the performed transfer learning based on one repetition gesture obtained more than 75% accuracy for both new subject and new gesture recognition, reached 90% accuracy when more than two repetitions were included and got an average accuracy of 91.18% for new gesture recognition in DB-a. In comparison, our proposed TL strategy obtained more than 88% accuracy for one repetition and reached 95% for more than two repetitions in both transfer learning tasks, and got an average accuracy of 94.7% in DB-a. Du [19] proposed a multi-stream AdaBn domain adaptation method based on CapgMyo. When adopting the adaptation method, the major voted recognition accuracy of new subjects rose from 26.3% to 35.1% for DB-c, while the accuracy of our proposed TL strategy was high than 75%. Ketyko [37] proposed a 2-stage recurrent neural networks. In their study, 50% of target subject data were used for second stage learning, and the evaluation was performed on the remaining target data. The accuracy of new subject recognition reached 91.1% and 65.1% for DB-c and Exercise A, respectively. In our study, the new subject recognition accuracy could reach 97.6% for DB-c and 79.4% for Exercise A by given 5 repetition target training data. Kim [38] presented a novel subject-transfer framework, and the target network training was based on single-trial sparse multi-channel sEMG. The experimental results exhibited the transfer learning ability of the approach, which improved the accuracy from 49.76% to 52.52%, while our proposed TL strategy improved the accuracy from 50% to 68.4%. Patricia [54] evaluated the four adaptive learning methods of new subject recognition and built a benchmark on NinaPro DB1. Best performance of the four methods achieved approximately 60% in Exercise A, while our proposed method got the best accuracy of 75.1%.

### 6.4. Comparison for HD-sEMG Database and Sparse Multi-Channel sEMG Database

For CapgMyo and NinaPro DB1, the proposed TL method reduces the training time by an average of three times on both databases. The average improvement exceeded 16% for new subject recognition and 7% for new gesture recognition when no more than three repetitions were given. Although the TL method is used, there is still a difference in the recognition accuracy of the two databases. The major voted accuracy of CapgMyo is about 30% higher than that of NinaPro DB1, and the instantaneous accuracy is approximately 10% higher. This is because CapgMyo has 72 more votes than NinaPro DB1. In addition, the increasing number of sEMG channels can bring more information, and also improve the recognition accuracy [55].

### 6.5. Prediction Delay

Instantaneous gesture recognition makes prediction base on the values of each sampling moment of the sEMG signal. The sampling rates of the CapgMyo database and NinaPro DB1 are 1000 Hz and 100 Hz, and the instantaneous prediction delays on the two databases are 1 ms and 10 ms, respectively. Similar to the result in the HD-sEMG classification study [19], the processing time of the gesture recognition network for instantaneous value of HD-sEMG and sparse multi-channel sEMG is about 0.5 ms on our workstation, which can meet the real-time requirements. The major voting scheme can improve the accuracy of prediction, and will also introduce prediction delay. However, a maximum time latency of 300 ms was recommended in [46]. In this paper, the voting numbers of the CapgMyo database and NinaPro DB1 are 100 and 28, and the corresponding major voted prediction delay are 100 ms and 280 ms, which are acceptable.

### 6.6. Limitations and Future Work

The limitations of this study and ideas for future work will now be discussed. First, although we have verified the validity of the proposed TL method, we use a simple convolutional neural network structure, which needs further improvement. Second, the target network training is still applied in a subject-dependent way. Third, our proposed TL strategy focuses on the transferability of the spatial information, while combining the temporal and spatial information may help boost the generalization performance. Future work will investigate the proper combination of temporal and spatial information of sEMG, and aim to build a general recognition system.

## 7. Conclusions

Based on the convolutional neural network structure and the spatial feature information hidden behind the instantaneous values of sEMG, a TL strategy for instantaneous gesture recognition has been proposed to improve the generalization ability of the target network for new subjects and new gestures. The proposed strategy first obtains the spatial feature extractor of the source network through pre-training, and then transfers the source spatial feature extractor to the target network. Finally, the fully connected layers of the target network were fine-tuned on the target database. High-density and sparse multi-channel sEMG databases were recruited to verify the validity of the TL method. The experimental results demonstrate that (1) the source spatial feature information can improve the accuracy of new subject and new gesture recognition, and; (2) the TL strategy can reduce the requirement for training time and data collection. The significant results verify the transferability and generalization ability of the spatial features in new subject and new gesture recognition, which indicates a way to enhance the generalization characteristic of gesture recognition systems.

## Figures and Tables

**Table 1:** Two-way ANOVA analysis results for the target network. * means the significant difference of *p* < 0.001.

Target		Factor	Mean Differernces and Sig. (*p*)		
Gesture Set			Instantaneous Accuracy	Major Voted Accuracy	Training Time
DB-c	Main	Training Strategy	<0.001 *	<0.001 *	<0.001 *
		Gesture Repetition	<0.001 *	<0.001 *	0.029
Exercise A	Main	Training Strategy	<0.001 *	<0.001 *	<0.001 *
		Gesture Repetition	<0.001 *	<0.001*	0.02
DB-a	Main	Training Strategy	<0.001 *	<0.001 *	<0.001 *
		Gesture Repetition	<0.001 *	<0.001 *	0.995
Exercise B	Main	Training Strategy	<0.001 *	<0.001 *	<0.001 *
		Gesture Repetition	<0.001 *	<0.001 *	0.584

**Table 2:** The average instantaneous accuracy (%) and average major voted accuracy (%) for new subject recognition.

Target Gesture Set		Gesture Repetitions						
		1	2	3	4	5	6	7
DB-c	Instantaneous accuracy	53.16	67.36	72.10	72.60	74.65	76.06	77.25
	Major voted accuracy	72.25	91.59	92.19	95.07	96.53	97.26	98.03
Exercise A	Instantaneous accuracy	55.46	59.55	64.51	66.24	68.06	67.84	70.41
	Major voted accuracy	59.29	64.33	70.80	73.74	73.68	74.01	75.53

**Table 3:** The comparison of training time(s) between the TL strategy and non-TL strategy for new subject recognition.

Target Gesture Set	Strategy	Gesture Repetitions						
		1	2	3	4	5	6	7
DB-c	TL	23.7 ± 6.94	29.8 ± 9.77	34.96 ± 12.95	47.65 ± 21.15	59.75 ± 27.63	76.7 ± 40.15	86.57 ± 43.02
	Non-TL	253.1 ± 2.1	176.63 ± 69.8	168.1 ± 65.3	124.1 ± 29.3	166.5 ± 90.35	148.8 ± 56.78	171.8 ± 76.25
Exercise A	TL	17.6 ± 6.31	18.9 ± 5.31	21.9 ± 7.57	24.7 ± 8.54	27.3 ± 8.83	27.3 ± 9.71	32.6 ± 11.7
	Non-TL	80.8 ± 26.1	80.4 ± 26.2	62.9 ± 29.7	62.6 ± 28.1	65.6 ± 25.5	72.9 ± 33.4	81.2 ± 37.1

**Table 4:** The average instantaneous accuracy (%) and the average major voted accuracy (%) for new gesture recognition.

Target Gesture Set		Gesture Repetitions						
		1	2	3	4	5	6	7
DB-a	Instantaneous accuracy	63.29	71.54	75.11	79.30	80.99	82.81	83.89
	Major voted accuracy	79.80	88.30	90.74	94.61	95.63	96.83	96.78
Exercise B	Instantaneous accuracy	47.60	52.50	56.18	56.82	59.95	60.04	60.07
	Major voted accuracy	51.30	56.91	62.09	63.21	64.74	64.33	65.43

**Table 5:** The comparison of training time(s) for TL strategy and non-TL strategy for new gesture recognition.

Target Gesture Set	Strategy	Gesture Repetitions						
		1	2	3	4	5	6	7
DB-a	TL	12.9 ± 6.62	18.4 ± 13.7	19.4 ± 8.76	22.7 ± 11.9	27.2 ± 11.9	29.5 ± 13.1	33 ± 18.5
	Non-TL	106 ± 57	101 ± 64.6	98.8 ± 65.6	90.5 ± 63.9	87.5 ± 56.2	76.1 ± 45.5	84.5 ± 61.4
Exercise B	TL	50.1 ± 11.8	46.9 ± 12	49.2 ± 10.6	41.6 ± 12.6	39.7 ± 11.3	40.1 ± 11.4	47 ± 22.8
	Non-TL	124 ± 14.9	115 ± 25	118 ± 20.4	120 ± 24.5	119 ± 30.1	127 ± 29.6	118 ± 30.5

## Data Availability

The NinaPro DB1 database is available at http://ninapro.hevs.ch (accessed on 21 January 2021). The CapgMyo database is available at http://zju-capg.org/research_en_electro_capgmyo.html (accessed on 21 January 2021).
